# Oxygen-enhanced MRI in patients with uterine cervical carcinoma: feasibility and treatment-associated changes in tumour oxygen-enhanced response

**DOI:** 10.3389/fonc.2026.1859532

**Published:** 2026-07-24

**Authors:** Anubhav Datta, Michael J. Dubec, Michael Berks, Ross A. Little, Lisa Barraclough, Kate Haslett, Nuria Porta, Prakash Manoharan, David L. Buckley, Catharine West, Ananya Choudhury, Peter Hoskin, James P. B. O’Connor

**Affiliations:** 1 Division of Cancer Sciences, University of Manchester, Manchester, United Kingdom; 2 Clinical Radiology, The Christie NHS Foundation Trust, Manchester, United Kingdom; 3 Christie Medical Physics and Engineering, The Christie NHS Foundation Trust, Manchester, United Kingdom; 4 Clinical Oncology, The Christie NHS Foundation Trust, Manchester, United Kingdom; 5 Clinical Trials and Statistics Unit, The Institute of Cancer Research, London, United Kingdom; 6 Biomedical Imaging, University of Leeds, Leeds, United Kingdom; 7 Clinical Oncology, Mount Vernon Cancer Centre, Northwood, United Kingdom; 8 Division of Radiotherapy and Imaging, The Institute of Cancer Research, London, United Kingdom

**Keywords:** biomarker, cervical cancer, hypoxia, MRI, radiotherapy

## Abstract

**Purpose:**

Tumour hypoxia causes resistance against radiotherapy and chemotherapy in locally advanced cervical cancer (LACC). Non-invasive magnetic resonance imaging (MRI) biomarkers have the potential to guide hypoxia-targeted therapy but require technical development and validation. This study evaluated the feasibility and early treatment response monitoring capabilities of oxygen-enhanced MRI (OE-MRI) in healthy female volunteers and patients with LACC.

**Methods:**

Healthy volunteers underwent OE-MRI to demonstrate the feasibility of the technique and to compare the onset, magnitude, and duration of change in longitudinal relaxation rate (Δ*R*_1_) for breathing either 100% oxygen or carbogen. Uterine cervix and body tissues were examined. Next, patients with biopsy-proven LACC underwent OE-MRI pre-treatment, and then, at 3 and 5 weeks into chemotherapy, it was combined with external beam radiotherapy.

**Results:**

Feasibility was demonstrated in 97% (31/32) of uterine tissue regions of interest, measured in 18 volunteers. Δ*R*_1_ values in the uterine cervix and body were similar across gas types with a cohort Δ*R*_1_ of between 0.026 and 0.033 s^−1^. In patients with LACC, significant increases in tumour Δ*R*_1_ were observed during treatment by week 3 (mean increase, 0.0143 s^−1^; 95% CI 0.0014 to 0.0273; *p* = 0.035). At week 5, six of eight evaluable patients showed a >50% treatment-associated increase in tumour Δ*R*_1_. Changes persisted in most patients to the end of treatment.

**Conclusion:**

This first dedicated OE-MRI study in cervical cancer shows technique feasibility in healthy volunteers and patients with LACC. Treatment-associated increases in tumour Δ*R*_1_ were demonstrated during chemoradiotherapy and likely reflect hypoxia modification. These findings support further evaluation of OE-MRI alongside independent hypoxia biomarkers in larger cervical cancer cohorts.

## Introduction

Uterine cervical cancer is a major global health problem. It is the fourth most frequent cancer in women worldwide, with an estimated 660,000 new cases in 2022 and approximately 350,000 deaths (1). Oxygen deficiency, termed hypoxia, is a hallmark of solid tumours that promotes tumour angiogenesis, genetic instability, and metastasis (2). The majority of patients present with locally advanced cervical cancer (LACC) that covers FIGO (International Federation of Gynecology and Obstetrics) stages IB_2_ to IVA. These patients are treated with combination radiotherapy and chemotherapy (3). Since inadequate tissue oxygenation mediates resistance to these therapies, tumour hypoxia is associated with reduced patient survival, independent of treatment option (4, 5). There is evidence in favour of hypoxia modification for locoregional control and benefitting overall survival (6). Furthermore, as hypoxia is a common feature of cancer but much less often present in healthy normal tissue, hypoxia-targeting therapies offer a high therapeutic index (7, 8).

Despite the strong rationale for its inclusion, hypoxia-targeted therapy is not standard treatment for LACC, unlike bladder cancer where the hypoxia-modifying treatment BCON is used for some indications (9). Many factors have contributed to the poor clinical translation of these therapies (6), but the lack of validated biomarkers to select the patients most likely to benefit from hypoxia-targeting treatments is a major factor. Invasive electrode measurements of oxygen (10), tissue biopsy-derived protein (11) or gene expression markers (12), and blood-based biomarkers (13) have a high biological specificity. However, these techniques do not offer any spatial information regarding whole tumour heterogeneity and require serial invasive sampling to track temporal changes. This limits their potential to personalise treatment pathways. It also hinders the development of hypoxia-driven strategies such as radiation dose escalation, radiation dose de-escalation, or addition of hypoxia-targeting therapies to standard of care (14).

Imaging offers a potential solution to this unmet need, by identifying, quantifying, and mapping the incidence of hypoxia prior to treatment and the duration of hypoxia persistence whilst on treatment. Positron emission tomography (PET) imaging is the most widely investigated method for imaging tumour hypoxia (15), but few studies have been performed in uterine cervical cancer. Some small exploratory studies show that imaging can detect cervical cancer hypoxia, but these have not yet been powered to explore if hypoxia imaging biomarkers relate to clinical outcome or other hypoxia biomarkers (16–18).

Magnetic resonance imaging (MRI) offers cheaper, more translational and more widely available alternative to image hypoxia, compared to PET techniques (19). One specific technique is oxygen-enhanced MRI (OE-MRI), which measures changes in longitudinal relaxation rate (Δ*R*_1_) (20) induced when subjects inhale air followed by either carbogen gas (95%–98% oxygen with 2%–5% carbon dioxide) or 100% oxygen (21). The technique can distinguish those regions of tumours that are normoxic from those that are hypoxic^22.^ Numerous studies have used both direct invasive pO_2_ histography and indirect (19) F-MRI histography and tissue pathology assays to biologically validate OE-MRI biomarkers in multiple mouse, rat, and rabbit models of cancer (22–25). OE-MRI has been translated in a range of clinical tumour populations (20, 24, 26–31) to demonstrate technique feasibility on both diagnostic scanners and an MR-Linac platform (32). Current translational interest is best exemplified by the ability of OE-MRI to identify, map, and quantify hypoxia modification, as seen in patients with lung cancer (25) and with oropharyngeal cancer (33), which may enable future selection of patients for altered radiotherapy strategies.

To date, OE-MRI has not been evaluated in a cohort of patients with uterine cervical carcinoma (27). This study had three aims; firstly, to develop OE-MRI for female pelvis imaging and translate it onto clinical MRI systems; secondly, to investigate if the technique was similar with oxygen and carbogen gas inhalation; and finally, to explore treatment-associated changes in tumour oxygen-enhanced response during chemoradiotherapy in patients with LACC.

## Materials and methods

### Healthy volunteer and patient cohorts

The “Biomarkers for Clinical Hypoxia Evaluation in Cervical Cancer” (BioCHECC) study (NCT05029258) was set up to prospectively recruit healthy volunteers and patients. Ethical approval was obtained (REC 20/NW/0377). Healthy volunteers and patients with LACC were included in this study. Inclusion criteria for all participants were women aged 18 years or more with no upper age limit. Exclusion for all participants was contraindication to MRI scanning.

Additional requirements for patient recruitment were biopsy-confirmed FIGO stages IB_2_ to IVA and planned to receive chemoradiotherapy with curative intent. Exclusion criteria were prior cancer diagnosis; prior hysterectomy, pelvic radiotherapy, or systemic chemotherapy; pregnancy or lactation; unsuitable for concurrent chemotherapy; and contraindication to one of the following: MRI, gadolinium-based contrast, or hyoscine-N-butylbromide.

### Patient treatment regimen

All patients received 45 Gy administered in 25 fractions (1.8 Gy per fraction; 5 weeks) of external beam radiotherapy (EBRT). The clinical target volume (CTV) was defined to include gross tumour volume, entire cervix, entire uterus, parametria, ovaries, and vagina depending on involvement, according to the EMBRACE protocol (34). Nodal CTV included the pelvic and para-aortic nodes. Patients also received two fractions of pulsed dose rate brachytherapy delivered with an intracavitary or a combined intracavitary/interstitial implant. MRI-guided adaptive brachytherapy (IGABT) was prescribed to deliver 40–45 Gy, calculated as equivalent dose in 2 Gy fractions (EQD2). This contributed to a total dose of 85–90 Gy EQD2 to the high-risk CTV and ≥60 Gy EQD2 to the intermediate-risk CTV. Single-agent concomitant cisplatin chemotherapy was given weekly at 40 mg/m (2).

### Imaging protocol and schedule

Imaging was acquired on either a diagnostic 1.5 T Philips Ingenia MR-RT (Philips Healthcare, Best, Netherlands) or a MR-Linac system (Elekta AB, Stockholm, Sweden) equipped with a Philips Marlin 1.5 T MRI scanner (Philips Healthcare, Best, Netherlands). Prior to image acquisition, all participants were asked to empty their urinary bladder using a “double-void” regime.

Healthy volunteers were recruited and imaged once. Patient imaging was acquired around the EBRT treatment schedule. Patients underwent imaging at baseline (pre-treatment), in the third week (W3; mid-EBRT), and in the fifth week (W5; end-EBRT) of treatment using a multi-parametric protocol.

All study participants underwent a T_2_-weighted (T_2_w) spin-echo scan to define pelvic anatomy, followed by three-dimensional (3D) T_1_ inversion-recovery turbo field echo (IRTFE) for T_1_ mapping and dynamic oxygen-enhanced (OE-MRI) measurements. A non-selective inversion pulse was utilised. All imaging was performed in the sagittal plane. Sagittal acquisition is routinely used in clinical practice and provides an excellent midline overview to evaluate organ spatial relationships. **Supplementary Table 1** displays the sequence details used in this study, which took up to 45 min of on-table scan time. Participants were positioned so that the uterine cervix was at the isocentre of the magnet. T_1_ mapping and OE-MRI acquisitions had a spatial resolution of 3.0 × 3.0 × 6.0 mm (3), a 128 × 128 matrix, and 40 contiguous slices.

OE-MRI consisted of an initial air phase (breathing medical air with 21% O_2_; during time points 0–25; 5 min) followed by a hyperoxic gas (time points 26–70; 9 min). Healthy volunteers inhaled either 100% oxygen or carbogen (here, CO_4_; mix of 98% oxygen and 2% carbon dioxide) as the hyperoxic gas, whereas all patients had 100% oxygen. Volunteers and patients had a final air phase breathing medical air with 21% O_2_ (time points 71–91; 4 min).

Medical air (21%) and 100% oxygen were delivered via the main hospital supply. Carbogen was stored and transported in a type AV cylinder (Material No. 294964-AV-PC; BOC Gases, Woking, UK) and was delivered using an O_2_ two-stage bullnose regulator (Catalogue ID 6070SH, Hitchen, UK) to normalise gas pressure. Gases were delivered to each participant using a tight-sealed, non-rebreathing Intersurgical EcoLite™ adult facemask (Intersurgical Ltd, UK). Switching between the gases—from medical air to either 100% O_2_ or carbogen gas—was controlled via the Low Flow Air-Oxygen Blender (Inspiration Healthcare, UK). An O_2_ hose assembled with British Standard (BS) and National Institute of Standards & Technology (NIST) probes was used to connect the gas outlet or regulator to the blender.

### Motion model in healthy volunteers

Regions of interest (ROIs) were assessed for bulk patient movement and physiological motion that could affect image alignment within a sequence or between sequences, since movements of the uterine body in the superior–inferior and anterior–posterior directions secondary to urinary bladder filling are well documented (35). Initial attempts at automated whole-image registration were unreliable and risked introducing local misregistration or interpolation-related artefact into the *R*_1_ time series. Therefore, an ROI-based motion tracking model was applied when deriving the Δ*R*_1_ parameter for the uterine body region in healthy volunteers (**Supplementary Figure 1**). As cervical displacement is typically less marked and rectal filling predominantly affects cervical position (35), no motion correction strategy was applied when analysing this region in healthy volunteers. Formal observer-repeatability assessment of motion tracking was not performed.

### Image analysis

Exploratory platform-stratified summaries and paired Bland–Altman analysis in healthy volunteers were additionally performed to assess potential platform effects in pelvic Δ*R*_1_ measurements. A previous study in oropharyngeal cancer by our group found no difference in the OE-MRI signal onset and magnitude between the diagnostic MR and MR Linac systems (32). Consequently, data acquired from the two scanners were pooled for analysis.

All ROIs were defined by a board-certified radiologist (AD, 7 years’ experience) and checked by a senior board-certified radiologist (JPBOC, 20 years’ experience). Image analysis was performed using MATLAB Release 2022a (Mathworks, Massachusetts, USA).

For OE-MRI healthy tissue assessments (both healthy volunteers and patients, an ROI measuring 7 rows × 7 columns of voxels (total of 49 voxels) was placed on a single sagittal slice and extended across two additional slices on either side, to create an ROI on five consecutive slices equivalent to 13.23 cm (3). This fixed-size approach was used to ensure consistent sampling of healthy tissues between participants. For OE-MRI tumour assessments, ROIs delineating the entire lesion outline were marked on T_2_w images using Jim 6 software (Xinapse Systems, Essex, UK). Whole-tumour delineation was used to capture treatment-associated change across the complete tumour volume. In all instances, voxel-wise model fitting was applied.

Signal data acquired using the IRTFE T_1_ mapping sequence at five inversion times (TI = 100, 500, 1,100, 2,000, and 4,300 ms) were used to estimate native T_1_ using the Equation 1 (36):

(1) $$\text{S}\left(\text{TI}\right)={\text{ S}}_{0}\left|1-2.\text{exp}\left(\frac{-\text{TI}}{T_{1}}\right)\right|\;$$

where S(TI) represents the signal intensities at each inversion time TI and S_0_ was the equilibrium signal. T_1_ and S_0_ were fit as free parameters via non-linear least squares fitting. *R*_1_ is the tissue longitudinal relaxation rate and is defined as the inverse of T_1_ (1/T_1_). The OE-MRI biomarker Δ*R*_1_ is defined as Equation 2 (37):

(2) $$\text{Δ}R_{1}\;=\;R_{1\left(\text{t}\right)}-\;R_{1\left(0\right)}$$

where *R*_1(t)_ is *R*_1_ whilst breathing hyperoxic gas (mean of time points 56–65) and *R*_1(0)_ is *R*_1_ on breathing air prior to the switch to hyperoxic gas (mean of time points 16–25). Subsequent assessment determined if the signal had returned to baseline, and *R*_1(t)_ is *R*_1_ whilst breathing air at the end of the gas challenge (mean time points 82–91; healthy volunteers only).

### Exploratory assessment of possible hypoxia modification

Previous clinical studies in patients with lung cancer (25) and with oropharyngeal cancer (33) have identified *R*_1_ changes of approximately 50% as indicating clinically relevant hypoxia modification. Based on this, a >50% relative increase in tumour Δ*R*_1_ from the pre-treatment value was defined *a priori* as an exploratory patient-level threshold. We considered exceeding this threshold as being evidence of possible hypoxia modification in patients in this study.

### Identification of volumetric response in cervical tumours

Tumour size was calculated in cm (3). In RECIST 1.1, a 30% change is considered to indicate reduction in a tumour in one dimension. Following previous work (38), we determined that a 3D equivalent estimate of tumour response is where a tumour measures 0.7 × 0.7 × 0.7 of its original volume = 0.343 (approximating the lesion as a sphere). Therefore, volume reduction of 1 − 0.343 = 0.657 (or 65.7%) was used to indicate lesion-specific “volumetric response”. This exploratory volumetric conversion assumes approximately isotropic tumour shrinkage and is not a formally validated volumetric RECIST criterion in cervical cancer.

### Statistical analyses

Statistical evaluation was supervised by a career statistician (NP, 15 years’ experience). Once derived, the imaging parameters were analysed as per Quantitative Imaging Biomarker Alliance (QIBA) recommendations (39). Analysis of variance (ANOVA) with *post hoc* Bonferroni correction was used to determine if Δ*R*_1_ was significant in each ROI (uterine cervix, uterine body, or tumour), both for individual participants and at the cohort level, where mean values of Δ*R*_1_ were used from each respective cohort (healthy volunteers or patients). Paired *t*-tests were used to compare any cohort sub-groups. Mean paired changes are reported with 95% confidence intervals. In all cases, significance was attributed when *p* < 0.05.

## Results

### Healthy volunteer cohorts and quality control

**Figure 1:**
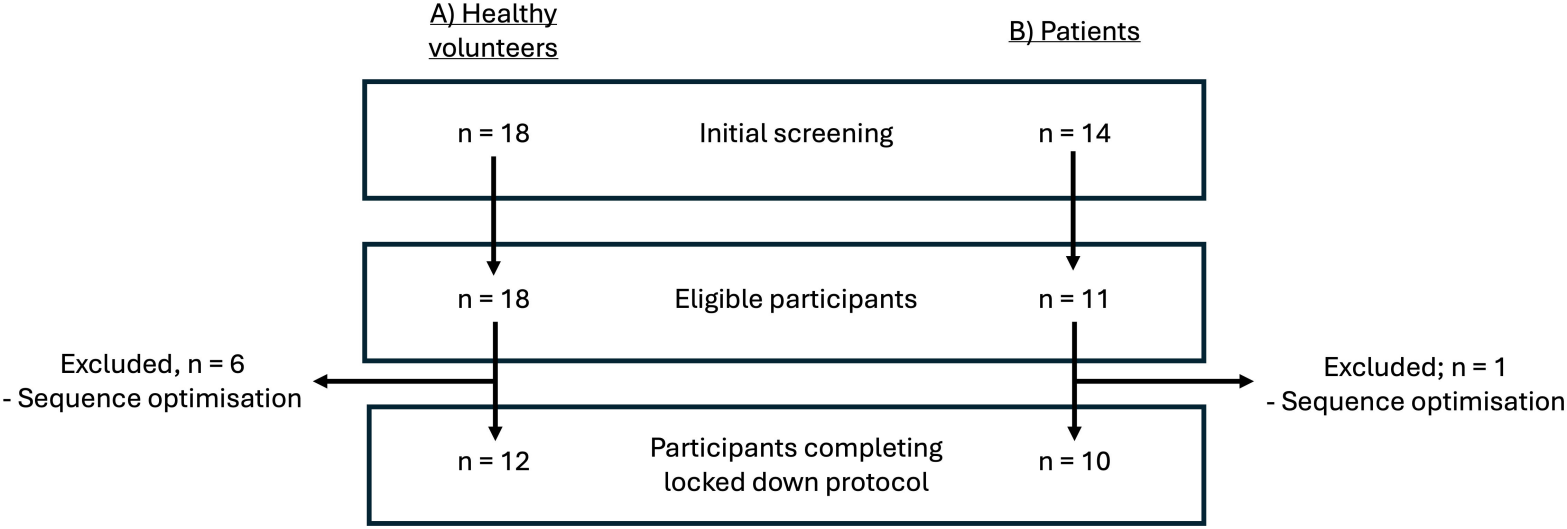
Participant flow in the study for **(a)** healthy volunteers and **(b)** patients.

Eighteen healthy volunteers were recruited (**Figure 1A**). Six healthy volunteers’ data were used to develop and finalise imaging protocols, which demonstrated good signal change induced by breathing 100% oxygen (**Supplementary Figure 2**), but not used for the main study analysis.

The mean age of the 12 volunteers used for the main study was 28 ± 5 years. Five healthy volunteers received OE-MRI with 100% oxygen as the hyperoxic gas and were imaged on the diagnostic MR system (these volunteers are denoted HV 6 to HV 10 in the display items). The same women had the same gas challenge on the MR Linac (these volunteers are denoted HV 6_MRL to HV 10_MRL in the display items). Motion tracking quality assurance was reviewed for each volunteer (**Supplementary Figure 3**).

Seven different healthy volunteers received OE-MRI with carbogen as the hyperoxic gas. Of these, three (HV 12 to HV 14) were imaged on the diagnostic MR and four (HV 15_MRL to HV 18_MRL) were imaged on the MR Linac. Motion tracking highlighted excessive uterine body motion in one volunteer (**Supplementary Figure 4**). These data were removed from cohort analysis.

Eleven patients with LACC were recruited (**Figure 1B**). The mean age of these women was 47 ± 18 years). Data from the one patient were used to confirm that the protocol was feasible and sensitive to gas challenge in a clinical tumour. The next 10 patients (denoted P2 to P11 in the display items) were included in the final clinical OE-MRI study. Data were not evaluable in P3 (tumour too small at the start of the study) and P9 (rectal gas artefact; **Supplementary Figure 6**), leaving eight patients for the final analysis.

### Δ*R*_1_ measurements in healthy tissues during and after hyperoxic gas challenge

**Figure 2:**
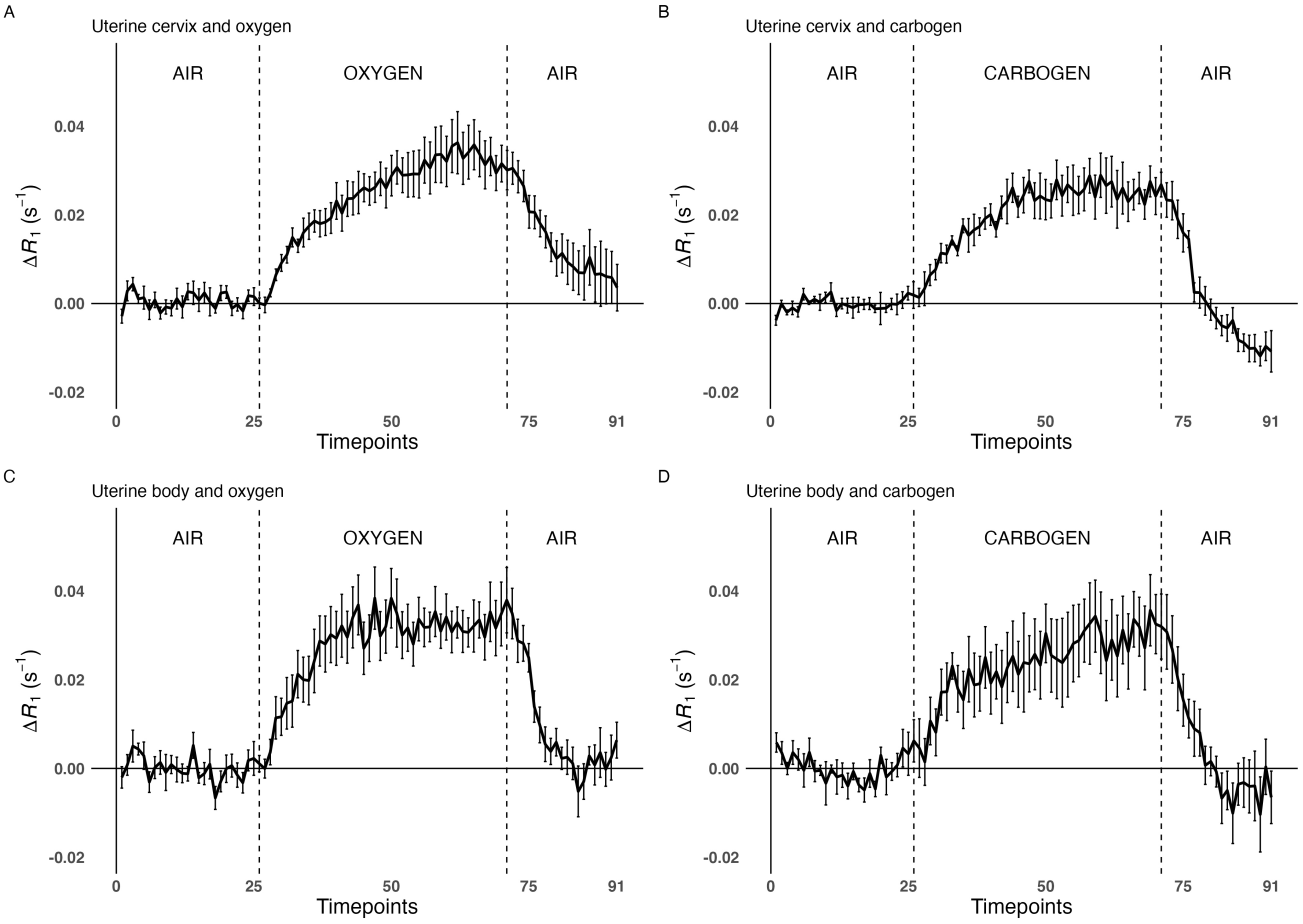
Oxygen-enhanced MRI (OE-MRI) signals are detectable in healthy uterine cervix **(A, B)** and uterine body **(C, D)** tissues. Cohort level Δ*R*_1_ time series, with error bars representing the standard error of the mean, were acquired from participants breathing oxygen (*n* = 10 scans) and carbogen (*n* = 6 scans). Note the similar onset slope and magnitude for oxygen and carbogen, but the relative overshoot of the Δ*R*_1_ when volunteers breathing carbogen switched back to final air breathing, relative to that seen in volunteers breathing the 100% oxygen protocol. The *x*-axis represents dynamic acquisitions (time points), where each time point corresponds to approximately 12–13.5 s depending on scanner platform. Thus, the full acquisition (91 time points) corresponds to approximately 18–20 min. Gas delivery phases (air, oxygen/carbogen, air) are indicated, with transitions at time points 25 and 70.

Data from 11 healthy volunteers were analysed to determine OE-MRI Δ*R*_1_ magnitude and range (**Table 1**). Cohort-level Δ*R*_1_ time series, for each tissue, are presented as the standard error of the mean (SEM) in **Figure 2**.

**Table 1:** Δ*R*_1_ values (s^−1^) in healthy volunteer tissue, for both uterine cervix and uterine body ROIs.

Participant	Uterine cervix Δ*R*_1_ (s^−1^)	Uterine body Δ*R*_1_ (s^−1^)
Inhaled 100% oxygen		
HV 6	0.030	0.024
HV 7	0.068	0.020
HV 8	0.027	0.019
HV 9	0.056	0.030
HV 10	0.028	0.032
HV 6_MRL	0.049	0.035
HV 7_MRL	0.004^NS^	0.037
HV 8_MRL	0.031	0.051
HV 9_MRL	0.013	0.060
HV 10_MRL	0.025	0.022
Inhaled carbogen (98% oxygen, 2% carbon dioxide)		
HV 12	0.010	0.014
HV 13	0.024	0.028
HV 14	0.020	0.009
HV 15_MRL	Motion corrupted	Motion corrupted
HV 16_MRL	0.032	0.048
HV 17_MRL	0.049	0.057
HV 18_MRL	0.022	0.024

Note that participant HV 15_MRL who inhaled carbogen had motion corruption in their images. All Δ*R*_1_ were significantly elevated on breathing hyperoxic gas except for HV 7_MRL uterine cervix data, which is denoted by NS (not significant).

*Individual subject analysis of air to hyperoxic gas ΔR_1_*: Measured Δ*R*_1_ were significant for all but one healthy volunteer scan ROI [HV 7 visit 1 scanned on the MRL; ROI for the uterine cervix region (Δ*R*_1_ = 0.004 s^−1^; *p* = 0.718)]. Notably, the uterine body for the same volunteer at the same visit exhibited significant change with inhalation of oxygen, indicating that this participant successfully complied with the gas inhalation protocol. In all, 31/32 (96.9%) of the uterine tissue ROIs demonstrated oxygen enhancement following hyperoxic gas breathing.

*Cohort analysis of air to hyperoxic gas ΔR_1_*: Mean cohort Δ*R*_1_ for the uterine cervix region in volunteers inhaling 100% oxygen was 0.033 s^−1^ (range, 0.004–0.068 s^−1^), and mean cohort Δ*R*_1_ for the uterine cervix region in volunteers inhaling carbogen was 0.026 s^−1^ (range, 0.0100–0.049 s^−1^). Mean cohort Δ*R*_1_ for the uterine body region in volunteers inhaling 100% oxygen was 0.033 s^−1^ (range, 0.019–0.060 s^−1^), and mean cohort Δ*R*_1_ for the uterine body region in volunteers inhaling carbogen was 0.030 s^−1^ (range, 0.009–0.057 s^−1^). No significant difference in mean cohort Δ*R*_1_ values were observed between the two hyperoxic gas cohorts.

*Cohort analysis of hyperoxia gas back to air ΔR_1_*: The initial and final air phases were examined (**Supplementary Table 2**). In the cohort inhaling 100% oxygen, the Δ*R*_1_ curve returned to baseline once the volunteers return to breathing air. There was no significant difference between the pairs of the Δ*R*_1_ values on the initial and final air phases for the uterine cervix or body regions (*p* = 0.229 and *p* = 0.587, respectively). In distinction, in the cohort inhaling carbogen, the Δ*R*_1_ curve overshot the baseline once the volunteers return to breathing air. The Δ*R*_1_ values on the final air phase were significantly less than those on the initial air phase for the uterine cervix (*p* = 0.027) but not the uterine body (*p* = 0.442).

Exploratory platform-stratified summaries and Bland–Altman analysis of the paired oxygen scans are provided in **Supplementary Table 3** and **Supplementary Figure 5**.

### Δ*R*_1_ increases during radiotherapy in cervical tumours indicating possible hypoxia modification

All patients underwent OE-MRI using 100% oxygen, chosen because the magnitude of change of Δ*R*_1_ was similar to that achieved with carbogen in healthy volunteers and 100% oxygen did not cause motion artefact in any volunteer data.

Clinicopathological data and Δ*R*_1_ measurements for the analysed patients are presented in **Table 2**. All patients had squamous cell cancers. Tumour volumes are shown in **Supplementary Table 4**. Six patients showed “volumetric response” (i.e., a reduction in tumour volume > 65.7%, the 3D equivalent of a one-dimensional response in RECIST) by W5, corresponding to a group mean reduction in tumour volume across all eight patients of 75.0% (with SD 14.5%).

**Table 2:** Clinicopathological data of prospective study patients.

ID	Age (years)	PS*	Stage	Grade^	LVSI†	Pelvic nodes	Responder (by volume)	Pre-treatment Δ*R*_1_ (s^−1^)	Mid-treatment Δ*R*_1_ (s^−1^)	End of treatment Δ*R*_1_ (s^−1^)
P2	73	0	IVA	3	Absent	Yes	Yes	0.008	0.012	0.010
P4	51	0	IIB	2	Absent	No	Yes	0.016	0.027	0.037
P5	30	0	IIIC1	3	Present	Yes	No	0.008	0.006	0.004
P6	37	1	IIB	3	Not known	No	Yes	0.006	0.045	0.024
P7	37	0	IIIC1	2	Not known	Yes	Yes	0.012	Not scanned	0.021
P8	46	0	IIB	3	Absent	No	No	0.008	0.015	0.022
P10	34	0	IIA	3	Present	No	Yes	0.007	0.030	0.067
P11	28	0	IVA	2	Present	No	Yes	0.030	0.050	0.024
Tumour µ ± SD								0.012 ± 0.008	0.027 ± 0.017	0.026 ± 0.020

MRI parameter Δ*R*_1_ values (s^−1^) for tumour assessments. All Δ*R*_1_ measurements showed a significant change from baseline when participants were switched from air to oxygen breathing, indicating successful oxygen inhalation.

*PS = Eastern Cooperative Oncology Group performance status scale (47).

^Grade 1: Well-differentiated (low grade); Grade 2: Moderately differentiated (intermediate grade); Grade 3: Poorly differentiated (high grade),

†LVSI = lymphovascular space invasion.

**Figure 3:**
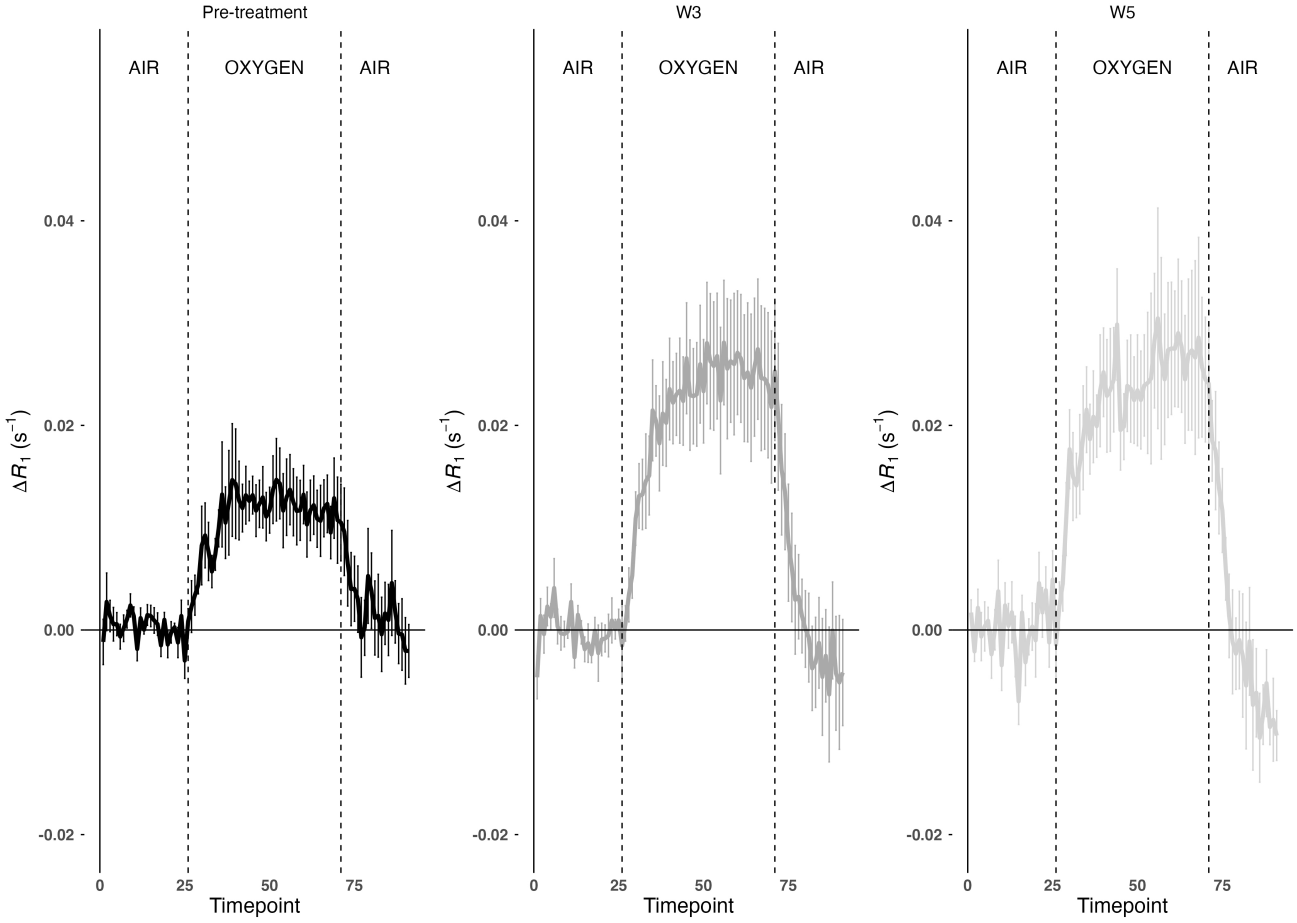
OE-MRI signals are detectable in tumours. Cohort level Δ*R*_1_ time series, with error bars representing the standard error of the mean, were created from pre-treatment, week 3, and week 5 data. Compared with pre-treatment, both W3 and W5 demonstrate increased Δ*R*_1_ during the oxygen-breathing window, consistent with greater oxygen-induced *R*_1_ enhancement later in treatment. The *x*-axis represents sequential dynamic acquisitions (time points), where each time point corresponds to approximately 12–13.5 s depending on scanner platform. Thus, the full acquisition (91 time points) corresponds to approximately 18–20 min. Gas delivery phases (air, oxygen, air) are indicated, with transitions at time points 25 and 70.

All tumours showed a significant oxygen enhancement measured by Δ*R*_1_ at all scan visits (pre-treatment, W3, and W5; **Supplementary Table 5**). The tumour mean Δ*R*_1_ parameter values ± SD increased during treatment at a cohort level: pre-treatment = 0.012 ± 0.008 s^−1^; W3 = 0.027 ± 0.017 s^−1^; W5 = 0.026 ± 0.020 s^−1^ (**Figure 3**).

Paired *t*-test between pre-treatment and W3 in seven evaluable patients showed a significant tumour Δ*R*_1_ increase of 0.0143 s^−1^ (95% CI 0.0014 to 0.0273; *p* = 0.035). Similarly, a tumour Δ*R*_1_ increase of 0.0139 s^−1^ (95% CI −0.0037 to 0.0315) was observed at W5 in all eight patients, but the overall cohort effect did not reach significance (*p* = 0.104; **Supplementary Table 6**).

**Figure 4:**
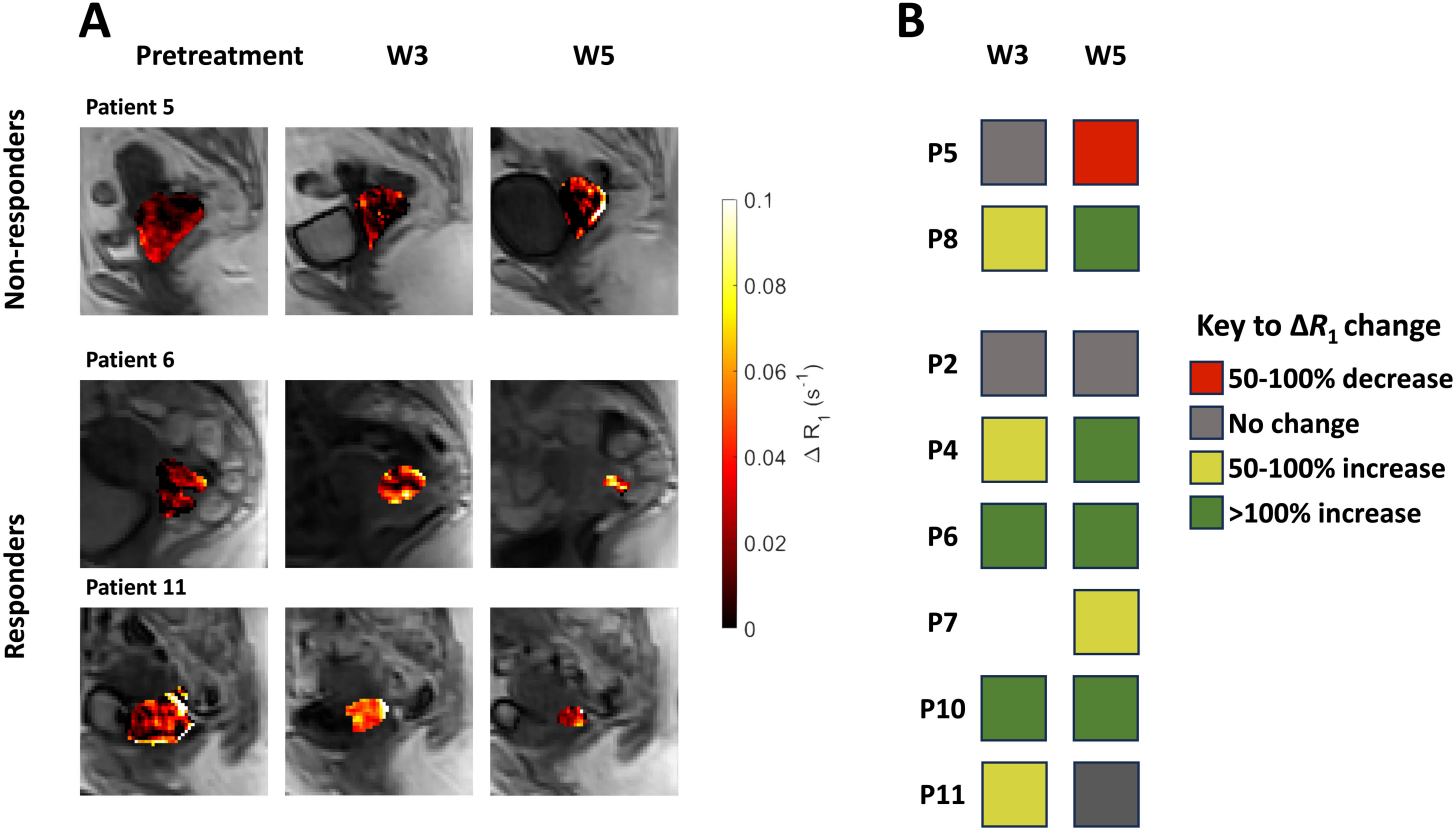
Patients were categorised initially as volume responders or non-responders (patients 5 and 8). Serial OE-MRI was then evaluated for each patient. **(A)** Example Δ*R*_1_ parameter maps at the mid-tumour level overlaid on the T_2_-weighted anatomical images. **(B)** Percentage changes in tumour Δ*R*_1_ categorised as potential increase in hypoxia (where Δ*R*_1<_ 50%), potential decrease in hypoxia (where Δ*R*_1_ > 50%), or no change exceeding either threshold. For comparison, occasions where Δ*R*_1_ > 100% are also shown.

Six of the eight patients had an increase in Δ*R*_1_ >50% during treatment. P2 showed an increase in Δ*R*_1_ during treatment but did not exceed the >50% threshold, whilst P5 showed no increase and was one of the two patients who were not volumetric responders by W5. Sample images for P5 and for two other patients who, in distinction, did show Δ*R*_1_ increases are shown (**Figure 4A**). The treatment-induced Δ*R*_1_ are also categorised by percentage change (**Figure 4B**). Classification according to alternative thresholds is shown in **Supplementary Table 7**, and baseline characteristics according to volumetric response status are shown in **Supplementary Table 8**.

## Discussion

MRI has the potential to provide non-invasive biomarkers of tumour oxygenation and hypoxia that could help personalise anticancer therapies. Previous studies have shown that OE-MRI can track hypoxia modification in patients with lung cancer (25) and oropharyngeal cancer (33). Three first-in-human findings are reported that may help advance OE-MRI in guiding treatment for patients with LACC.

Firstly, technique feasibility of OE-MRI in the female pelvis was demonstrated. Data show that the finalised protocol was well tolerated by all participants. No adverse events were reported. The mean cohort Δ*R*_1_ in the uterine body and cervix was documented, along with the range of values for healthy volunteers inhaling either 100% oxygen or carbogen gas. 100% oxygen breathing and carbogen breathing induced equivalent Δ*R*_1_ onset and magnitude, in keeping with previous studies (21, 40). Exploratory analyses identified possible platform-dependent differences in pelvic Δ*R*_1_ measurements; however, the diagnostic MR to MR-Linac transferability remains to be established in larger paired datasets.

Secondly, a difference in the physiological effects induced by carbogen compared to 100% oxygen in the uterine cervix was identified. The literature reports vasoconstriction and reduced blood flow following 100% oxygen inhalation (41, 42), which is the main reason why carbogen gas inhalation has been preferred traditionally in the radiotherapy setting (43, 44). Carbogen is believed to counteract the oxygen-induced vasoconstriction (45), though the optimal gas mixture (oxygen-to-carbon dioxide ratio) for treatment is not defined (46). In this study, differences in tissue oxygenation—as measured by OE-MRI—were detected in the final air phase, with volunteers who had breathed carbogen exhibiting an overshoot in the *R*_1_ return to baseline values. Further work is required to elucidate the relevance of this finding, but this does suggest that carbogen induces a more complex physiological effect in healthy tissues compared to those of 100% oxygen.

Thirdly, OE-MRI was examined in patients with cervical cancer. Previously, one study from our group had reported technique feasibility in two patients with cervical cancer amongst a wider cohort of mixed solid tumours (27). This paper reports the first study specifically designed to evaluate the potential of OE-MRI in cervical cancer. Six of the eight patients had Δ*R*_1_ > 50% during treatment—a possible hypoxia modification threshold based on literature precedent—with nearly all exhibiting this change by W3 and persisting to W5. These findings were consistent with other human OE-MRI studies of lung cancer (25) and oropharyngeal cancer (33). This translated into a cohort effect at W3. Two patients did not exceed the >50% Δ*R*_1_ threshold in their tumour: one subsequently demonstrated “volumetric response”, whereas the other did not.

Study limitations exist. All evaluable tumours were squamous cell carcinomas, which limits generalisability. Relatively small numbers of participants, especially only two non-responders, were included in this study. This precluded assessment of whether tumour Δ*R*_1_ predicts anatomical response. Of note, the >50% Δ*R*_1_ threshold was derived from other tumour types and has not been validated in cervical cancer. The small numbers are justified by the exploratory nature of the study and by using healthy volunteers to demonstrate technique feasibility first, before translating to an initial clinical cohort. Motion within the pelvis did lead to some data loss, but this had a negligible impact on the main study findings. Independent hypoxia validation (e.g., immunohistochemical markers, hypoxia gene signatures, pO_2_ electrode measurements, or hypoxia PET imaging) was not performed. Possible platform-dependent differences in pelvic Δ*R*_1_ measurements and the absence of formal observer-repeatability assessment are additional technical limitations. Although OE-MRI validation has been demonstrated in preclinical work, clinical validation remains to be established.

Data reported here provide initial evidence that OE-MRI is a viable technique for quantifying and mapping oxygen-enhanced response in patients with LACC. The data enable the calculation of effect size that can help power subsequent investigation using oxygen-induced Δ*R*_1_ as a potential biomarker of hypoxia modification in this patient population. Ideally, this would be alongside independent hypoxia validation. The longer-term aim is to translate OE-MRI to help personalise treatment strategies for cervical cancer based on tumour hypoxia and its modification during radiation-based therapy.

## Data Availability

The raw data supporting the conclusions of this article will be made available by the authors, without undue reservation.
