# Supplementary material for: Oxygen-enhanced MRI in patients with uterine cervical carcinoma: feasibility and treatment-associated changes in tumour oxygen-enhanced response
Source: Front Oncol. 2026 Jul 24;16:1859532. doi: 10.3389/fonc.2026.1859532 (PMC13447193; doi:10.3389/fonc.2026.1859532)
Supplement: Supplementary file 1 [file SupplementaryFile1.docx]

**Supplementary Tables and Figures**

**Supplementary Table S1:** Summary of MRI acquisition parameters used in the study.

|  | Diagnostic MRI | MR Linac |  |
| --- | --- | --- | --- |
|  |  |  |  |
| Sequence | **3D IR-TFE (T_1_ mapping)** | **3D IR-TFE (T_1_ mapping)** |  |
| TR | 2.2 ms | 2.3 ms |  |
| TE | 0.66 ms | 0.75 ms |  |
| TI | 100, 500, 1100, 2000, 4300 ms | 100, 500, 1100, 2000, 4300 ms |  |
| ⍺ | 4° | 6° |  |
|  |  |  |  |
| Sequence | **3D IR-TFE (OE-MRI)** | **3D IR-TFE (OE-MRI)** |  |
| TR | 2.2 ms | 2.3 ms |  |
| TE | 0.66 ms | 0.75 ms |  |
| TI | 1100 ms | 1100 ms |  |
| ⍺ | 4° | 6° |  |
| Number of measurements | 91 | 91 |  |
| Temporal resolution | 12 s | 13.5 s |  |
|  |  |  |  |

**Supplementary Table S2:** Δ*R*_1_ value (s^-1^) at air phases 1 and 2 in healthy volunteer tissue, for both uterine cervix and uterine body ROIs.

|  | Uterine cervix | | Uterine body | |
| --- | --- | --- | --- | --- |
|  | Air phase 1 (mean of time points 16 – 25) | Air phase 2 (mean time points 82 – 91) | Air phase 1 (mean of time points 16 – 25) | Air phase 2 (mean time points 82 – 91) |
| *Inhale 100% oxygen* | | | | |
| HV6 | -0.001 | 0.000 | -0.003 | -0.008 |
| HV7 | 0.001 | 0.033 | 0.000 | 0.006 |
| HV8 | 0.005 | 0.012 | 0.003 | 0.018 |
| HV9 | 0.000 | 0.012 | 0.000 | 0.003 |
| HV10 | -0.001 | -0.003 | 0.000 | -0.010 |
| HV6_MRL | -0.003 | -0.005 | -0.001 | -0.002 |
| HV7_MRL | 0.007 | 0.041 | -0.007 | 0.023 |
| HV8_MRL | -0.001 | 0.000 | -0.004 | -0.007 |
| HV9_MRL | 0.000 | -0.006 | 0.002 | -0.007 |
| HV10_MRL | 0.000 | -0.013 | 0.000 | -0.004 |
| *Inhale carbogen (98% oxygen; 2% carbon dioxide)* | | | | |
| HV12 | 0.003 | -0.016 | -0.002 | -0.016 |
| HV13 | -0.001 | -0.008 | -0.002 | -0.011 |
| HV14 | 0.002 | -0.003 | -0.001 | -0.010 |
| HV15_MRL | motion corrupted | | | |
| HV16_MRL | 0.000 | -0.003 | 0.003 | -0.009 |
| HV17_MRL | -0.002 | -0.016 | -0.001 | -0.010 |
| HV18_MRL | -0.002 | -0.005 | -0.001 | 0.023 |

**Supplementary Table S3: Exploratory assessment of scanner-platform effects on healthy-volunteer pelvic** Δ*R*_1_ **measurements.** The Bland–Altman analysis was restricted to healthy volunteers undergoing paired oxygen-enhanced MRI on both scanner platforms. The carbogen cohorts were unpaired and were therefore summarised descriptively only.

**A. Platform-stratified** Δ*R*_1_ **summaries**

| Hyperoxic gas | Tissue region | Scanner platform | n | Mean Δ*R*_1_ (s⁻¹) | Range (s⁻¹) |
| --- | --- | --- | --- | --- | --- |
| Oxygen | Uterine cervix | Diagnostic MR | 5 | 0.0418 | 0.0271 to 0.0683 |
| Oxygen | Uterine cervix | MR-Linac | 5 | 0.0241 | 0.0040 to 0.0485 |
| Oxygen | Uterine body | Diagnostic MR | 5 | 0.0252 | 0.0193 to 0.0324 |
| Oxygen | Uterine body | MR-Linac | 5 | 0.0410 | 0.0220 to 0.0597 |
| Carbogen | Uterine cervix | Diagnostic MR | 3 | 0.0178 | 0.0100 to 0.0236 |
| Carbogen | Uterine cervix | MR-Linac | 3 | 0.0341 | 0.0216 to 0.0485 |
| Carbogen | Uterine body | Diagnostic MR | 3 | 0.0170 | 0.0088 to 0.0280 |
| Carbogen | Uterine body | MR-Linac | 3 | 0.0428 | 0.0239 to 0.0566 |

**B. Bland–Altman agreement analysis for paired oxygen scans acquired on diagnostic MR and MR-Linac**

| Tissue region | Number of paired volunteers | Mean difference: MR-Linac − Diagnostic MR Δ*R*_1_ (s⁻¹) | SD of differences (s⁻¹) | Lower 95% limit of agreement (s⁻¹) | Upper 95% limit of agreement (s⁻¹) |
| --- | --- | --- | --- | --- | --- |
| Uterine cervix | 5 | −0.0176 | 0.0347 | −0.0857 | 0.0504 |
| Uterine body | 5 | 0.0158 | 0.0169 | −0.0174 | 0.0489 |

**Supplementary Table S4:** Tumour volume (cm^3^) at different treatment timepoints.

| Patient ID | Visit | Volume (cm³) |
| --- | --- | --- |
| 2 | pre | 77.5 |
| 4 | pre | 24.4 |
| 5 | pre | 304.8 |
| 6 | pre | 84.4 |
| 7 | pre | 536.8 |
| 8 | pre | 118.3 |
| 10 | pre | 27.9 |
| 11 | pre | 128.7 |
| 2 | W3 | 36.5 |
| 4 | W3 | 5.5 |
| 5 | W3 | 195.8 |
| 6 | W3 | 55.3 |
| 7 | W3 | not scanned |
| 8 | W3 | 55.9 |
| 10 | W3 | 12.5 |
| 11 | W3 | 57.5 |
| 2 | W5 | 17.1 |
| 4 | W5 | 5.5 |
| 5 | W5 | 109.4 |
| 6 | W5 | 7.6 |
| 7 | W5 | 74.9 |
| 8 | W5 | 65.2 |
| 10 | W5 | 6.4 |
| 11 | W5 | 23.5 |

**Supplementary Table S5:** Tumour Δ*R*_1_ (s^-1^) and Student’s t-test *p* values for within-visit oxygen enhancement. The p values assess the change in tumour R1 between the baseline air-breathing phase and the oxygen-breathing phase at each individual scan visit; they do not represent longitudinal comparisons between treatment visits.

| Patient ID | Visit | Δ*R*₁ (s⁻¹) | P value |
| --- | --- | --- | --- |
| 2 | pre | 0.008 | 5.1 × 10⁻⁸ |
| 4 | pre | 0.016 | 1.4 × 10⁻⁸ |
| 5 | pre | 0.008 | 3.0 × 10⁻⁸ |
| 6 | pre | 0.006 | 9.1 × 10⁻⁵ |
| 7 | pre | 0.012 | 6.6 × 10⁻¹⁷ |
| 8 | pre | 0.008 | 5.2 × 10⁻⁸ |
| 10 | pre | 0.007 | 0.015 |
| 11 | pre | 0.030 | 9.0 × 10⁻¹⁸ |
| 2 | W3 | 0.012 | 1.8 × 10⁻⁵ |
| 4 | W3 | 0.027 | 2.1 × 10⁻¹⁰ |
| 5 | W3 | 0.006 | 3.7 × 10⁻⁶ |
| 6 | W3 | 0.045 | 6.0 × 10⁻¹⁹ |
| 7 | W3 | not scanned |  |
| 8 | W3 | 0.015 | 2.6 × 10⁻⁸ |
| 10 | W3 | 0.030 | 1.7 × 10⁻¹¹ |
| 11 | W3 | 0.050 | 1.0 × 10⁻²¹ |
| 2 | W5 | 0.010 | 0.001 |
| 4 | W5 | 0.037 | 2.3 × 10⁻⁷ |
| 5 | W5 | 0.004 | 0.003 |
| 6 | W5 | 0.024 | 1.9 × 10⁻⁵ |
| 7 | W5 | 0.021 | 6.2 × 10⁻¹⁰ |
| 8 | W5 | 0.022 | 1.6 × 10⁻¹¹ |
| 10 | W5 | 0.067 | 2.7 × 10⁻¹⁰ |
| 11 | W5 | 0.024 | 4.9 × 10⁻⁸ |

**Supplementary Table S6:** Treatment-associated change in tumour oxygen-enhanced response (change in Δ*R*_1_) was defined as the difference between the on-treatment tumour Δ*R*_1_ value and the pre-treatment tumour Δ*R*_1_ value. Comparisons were performed using paired t-tests for patients with evaluable imaging at both visits.

| Comparison | Number of paired patients | Mean change in Δ*R*_1_ between visits (s⁻¹) | 95% CI (s⁻¹) | Paired t-test p value |
| --- | --- | --- | --- | --- |
| Week 3 − pre-treatment | 7 | 0.0143 | 0.0014 to 0.0273 | 0.035 |
| Week 5 − pre-treatment | 8 | 0.0139 | −0.0037 to 0.0315 | 0.104 |

Abbreviations: CI, confidence interval; Δ*R*_1_, change in longitudinal relaxation rate induced by oxygen breathing within an individual scan visit

**Supplementary Table S7: Sensitivity analysis of thresholds for treatment-associated percentage change in tumour** Δ*R*_1_**.** For each patient, the maximum percentage increase in tumour Δ*R*_1_ at either on-treatment assessment was calculated relative to the pre-treatment Δ*R*_1_ value. Thresholds are exploratory and were assessed to demonstrate the effect of cut-off selection on patient classification.

| Threshold for maximum percentage increase in tumour Δ*R*_1_ | Patients exceeding threshold | Number exceeding threshold | Volumetric responders exceeding threshold | Volumetric non-responders exceeding threshold |
| --- | --- | --- | --- | --- |
| >30% | P2, P4, P6, P7, P8, P10, P11 | 7/8 | 6/6 | 1/2 |
| >50% | P4, P6, P7, P8, P10, P11 | 6/8 | 5/6 | 1/2 |
| >75% | P4, P6, P8, P10 | 4/8 | 3/6 | 1/2 |

Volumetric response was defined as a reduction in tumour volume of >65.7% by week 5.

**Supplementary Table S8: Baseline characteristics of patients according to volumetric response status.** Volumetric response was defined as a reduction in tumour volume of >65.7% by week 5. Data are presented descriptively because only two patients did not meet the volumetric response criterion.

| Volume response status | Patient ID | Age (years) | FIGO stage | Pre-treatment tumour volume (cm³) | Pre-treatment tumour Δ*R*_1_ (s⁻¹) |
| --- | --- | --- | --- | --- | --- |
| Non-responder | P5 | 30 | IIIC1 | 304.8 | 0.008 |
| Non-responder | P8 | 46 | IIB | 118.3 | 0.008 |
| Responder | P2 | 73 | IVA | 77.5 | 0.008 |
| Responder | P4 | 51 | IIB | 24.4 | 0.016 |
| Responder | P6 | 37 | IIB | 84.4 | 0.006 |
| Responder | P7 | 37 | IIIC1 | 536.8 | 0.012 |
| Responder | P10 | 34 | IIA | 27.9 | 0.007 |
| Responder | P11 | 28 | IVA | 128.7 | 0.030 |

**Supplementary Figure S1:** Application of a motion tracking quality assurance step. Five ROIs were placed in a defined location at specified image timepoints in the dynamic series (timepoints 1, 31, 61 and 91), and ROIs were interpolated for the intervening timepoints. A ‘static’ ROI model was also derived from the first timepoint ROI for uterine body assessments.

**Supplementary Figure S2:** Early examples of healthy volunteer pelvic imaging using OE-MRI sequences in development. Artefact (*) from areas of signal drop out in (a) and oxygen tubing interposed between participant and the anterior body coil in (b). Initial assessment of the uterine body (c) in the finalised protocol a higher quality OE-MRI scan resulted in (d) a signal time-series showing a clear signal time-series which shows a change in signal following 100% oxygen delivery.

**Supplementary Figure S3:** *R*_1_ timeseries in the uterine body (UB) for healthy volunteers (HV) participants inhaling oxygen and scanned on the Diagnostic MR (left hand column) and MR Linac (right hand column). Data were analysed using motion tracked (red) and static (blue) regions of interest (ROIs). Note the y-axis varies in scale to fit each HV data. The ‘motion tracked’ UB *R*_1_ (red line) compared to ‘static’ *R*_1_ (blue line) showed a) little variation in baseline value compared to peak enhancement calculation, and b) a more consistent decrease in *R*_1_ when participants were switched from oxygen to medical air breathing. The UB ‘motion tracked’ *R*_1_ timeseries (red line) corrected motion-induced signal corruption for all visits.

HV 6

HV 7

HV 8

HV 9

HV 10

HV 6_MRL

HV 7_MRL

HV 8_MRL

HV 9_MRL

HV 10_MRL

**Diagnostic MR**

**MR Linac**

**Supplementary Figure S4:** *R*_1_ timeseries in the uterine body (UB) for healthy volunteers (HV) participants inhaling carbogen and scanned on the Diagnostic MR (left hand column) and MR Linac (right hand column). Data were analysed using motion tracked (red) and static (blue) regions of interest (ROIs). Note the y-axis varies in scale to fit each HV data. The ‘motion tracked’ UB *R*_1_ (red line) compared to ‘static’ *R*_1_ (blue line) showed a) little variation in baseline value compared to peak enhancement calculation, and b) a more consistent decrease in *R*_1_ when participants were switched from carbogen to medical air breathing. Detailed analysis showed that the UB ‘motion tracked’ *R*_1_ timeseries (red line) was unable to correct motion induced signal corruption in one patient: HV 15.

HV 12

HV 13

HV 14

HV 15

HV 16

HV 17

HV 18

**Diagnostic MR**

**MR Linac**

**Supplementary Figure S5: Bland–Altman analysis of pelvic** Δ*R*_1_ **measurements acquired on diagnostic MR and MR-Linac in healthy volunteers undergoing paired oxygen challenge imaging.** Bland–Altman plots are shown for the uterine cervix (A) and uterine body (B). Differences are defined as MR-Linac Δ*R*_1_ minus diagnostic MR Δ*R*_1_. Solid horizontal lines represent the mean difference between platforms and dashed horizontal lines represent the 95% limits of agreement. The analysis includes the five healthy volunteers who underwent paired oxygen-enhanced imaging on both scanner platforms.

**Diagnostic MR**

**MR Linac**

**Supplementary Figure S6:** Two patients omitted from further analysis. Patient 3 had a small tumour (white arrows; patient 9 had a larger tumour which is highlighted for comparison). Patient 9 had excessive rectal motion and gas (rectum highlighted by white star) which disrupted the *R*_1_ signal.
